# Characterization of a proximal Sp1 response element in the mouse *Dlk2 *gene promoter

**DOI:** 10.1186/1471-2199-12-52

**Published:** 2011-12-20

**Authors:** Samuel Rivero, Almudena Ruiz-García, María JM Díaz-Guerra, Jorge Laborda, José J García-Ramírez

**Affiliations:** 1 Facultad de Medicina/Centro Regional de Investigaciones Biomédicas (CRIB), Universidad de Castilla-La Mancha. Calle Almansa 14. 02006 Albacete. Spain; 2 Samuel Rivero. Molecular Pathology Section. Laboratory of Immunogenetics. National Institute of Allergy and Infectious Diseases, NIH. Twinbrook Building I, Room 1524. 5640 Fisher Lane. Rockville, MD 20852, USA

## Abstract

**Background:**

DLK2 is an EGF-like membrane protein, closely related to DLK1, which is involved in adipogenesis. Both proteins interact with the NOTCH1 receptor and are able to modulate its activation. The expression of the gene *Dlk2 *is coordinated with that of *Dlk1 *in several tissues and cell lines. Unlike *Dlk1*, the mouse *Dlk2 *gene and its locus at chromosome 17 are not fully characterized.

**Results:**

The goal of this work was the characterization of *Dlk2 *mRNA, as well as the analysis of the mechanisms that control its basal transcription. First, we analyzed the *Dlk2 *transcripts expressed by several mouse cells lines and tissues, and mapped the transcription start site by 5' Rapid Amplification of cDNA Ends. *In silico *analysis revealed that *Dlk2 *possesses a TATA-less promoter containing minimal promoter elements associated with a CpG island, and sequences for Inr and DPE elements. Besides, it possesses six GC-boxes, considered as consensus sites for the transcription factor Sp1. Indeed, we report that Sp1 directly binds to the *Dlk2 *promoter, activates its transcription, and regulates its level of expression.

**Conclusions:**

Our results provide the first characterization of *Dlk2 *transcripts, map the location of the *Dlk2 *core promoter, and show the role of Sp1 as a key regulator of *Dlk2 *transcription, providing new insights into the molecular mechanisms that contribute to the expression of the *Dlk2 *gene.

## Background

*Dlk2 *encodes for a transmembrane glycoprotein with six epidermal growth factor-like (EGF-like) motifs in the extracellular domain, a single transmembrane domain and a short intracellular tail. These features place DLK2 as a member of the EGF-like family of proteins, in which NOTCH receptors and their ligands are included [1]. The proteins of this family mediate protein-protein interactions through their EGF-like repeats, modulating cell fate differentiation in numerous cell types. DLK2 shares most of its structural features with DLK1, with the highest homology located at the EGF-like domains. DLK1 participates in several differentiation processes, including adipogenesis [1-6], differentiation of hepatocytes [7,8], hematopoiesis [2,9-14], osteogenesis [15-17], adrenal gland and neuroendocrine cell differentiation [18-23], peripheral and central nervous system differentiation [22,24], growth arrest, and increased malignancy of undifferentiated tumors [21,25-27]. DLK1 has also been reported to participate in the wound healing process [28]. DLK2 has been shown to participate also in adipogenesis [1], but its role in other differentiation processes is yet unknown.

*Dlk2 *expression can be detected in several adult mouse tissues, showing a more widespread pattern of expression than *Dlk1. Dlk2 *is highly expressed in lung, brain, adipose tissue, testicles, adult liver, placenta, ovaries and thymus [1]. Little is known about the regulation of *Dlk2 *expression, although it seems clear that the expression of *Dlk1 *and *Dlk2 *appears to be coordinated in some instances *in vitro*. Thus, their expression levels in response to cell confluence vary in opposite directions. Interestingly, when the expression level of one homolog is modified in one direction, the enforced change exerts an opposite effect on the expression level of the other, both in 3T3-L1 and C3H10T1/2 cells [1]. That seemingly coordinated expression appears to occur also during tissue development: along mouse embryogenesis and postnatal growth, *Dlk1 *is highly expressed during the development of fetal liver, when no expression of *Dlk2 *is detected; *Dlk2 *expression in liver can only be detected 16 days after birth [1]. All these data suggest the likely existence of coordinated control mechanisms for *Dlk1 *and *Dlk2 *gene expression.

Previous to this work, in the UCSC genome browser (http://genome.ucsc.edu), three full-length transcripts, BC118057, BC122518, and BC019431, had been assigned to *Dlk2*. The main differences among those *Dlk2 *transcripts are restricted to the 5' end of the mRNA, with most of the transcripts being identical in the majority of mRNA's 3' regions. To the best of our knowledge, experimental support regarding any of the three abovementioned transcripts is lacking, excluding a few publications regarding the role of *Dlk2 *[1,29]. In this paper, we describe the first experimental characterization of *Dlk2 *transcription, showing that only one out of the three predicted transcripts, BC019431, could be detected in all the mouse cell lines and tissues analyzed. We have also mapped the transcription initiation site, which correlates with the abovementioned transcript, although with 14 additional bp at the 5' end. The *Dlk2 *core promoter is located within a CpG island extending beyond the transcription start site (TSS). Bioinformatics analysis showed the presence of two core promoter elements, the Initiator Element (Inr), and the Downstream Promoter Element (DPE), which have been described as necessary for basal transcription in other genes. Finally, as it is characteristic of TATA-less promoters with an Inr element, we have shown that Sp1, a member of the Sp/KLF family of zinc finger transcription factors that recognize GC/GT boxes present in many GC-rich promoters, is able to bind to the *Dlk2 *promoter and control *Dlk2 *basal transcription.

## Results

### Characterization of *Dlk2 *mRNA

**Figure 1:**
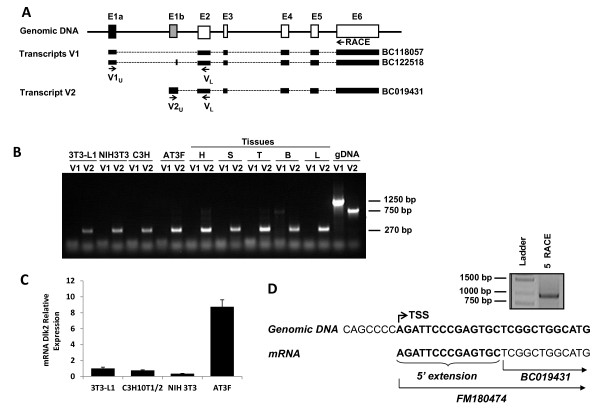
**Characterization of the *Dlk2 *transcription start site**. A) Schematic exon-intron distribution of *Dlk2 *cDNAs putatively assigned to *Dlk2*. The arrows indicate the position of oligonucleotides used to specifically amplify *Dlk2 *transcripts. B) RT-PCR of *Dlk2 *transcripts with the following templates: cDNAs from 3T3-L1, NIH3T3, C3H10T1/2, or AT3F cells; cDNAs from heart (H), spleen (S), testicles (T), brain (B) and lung (L) of adult 129/C57BL6 mice; or genomic DNA (gDNA). Two PCR reactions were performed with each template, one to amplify variant 1 transcript (V1) and another to amplify variant 2 (V2). The sizes of the expected amplified DNA fragments are shown on the right. C) Analysis of *Dlk2 *mRNA expression levels in 3T3-L1, NIH3T3, C3H10T1/2 and AT3F cell lines by RT-qPCR. mRNA levels were referred to the expression level of phosphoriboprotein P0, which was used as an internal control. D) Experimental determination of *Dlk2 *TSS by 5' RACE. RNA from AT3F cells was used for 5' RACE amplification, using a specific reverse primer located within the sixth exon, at position +693 from the translation initiation codon (ATG), indicated by an arrow in the upper pannel. The amplified PCR products were cloned into the pCR2.1 vector, and twenty individual clones were sequenced. The 5' region sequences of genomic DNA and cDNA clone BC091431 are shown, including the 14 additional bases of the newly described clone FM180474.

Mouse *Dlk2 *is a gene located at chromosome 17 (position 46434370-46440220), containing six exons. Previous to this work, three mRNAs have been associated to *Dlk2 *in GenBank: [GenBank: BC118057, BC122518, and BC019431] (http://genome.ucsc.edu) (Figure 1A). BC118057 and BC122518 show almost the same exon-intron distribution, just differing in the second exon of BC122518, which is not present in BC118057. BC019431 share the last four exons with the former two mRNAs. However, the transcription start site and the splicing sites for the first and second exons are different between the two sets of mRNAs clones, BC118057/BC122518 and BC019431. Surprisingly, BC019431 has been considered as a *chimeric *cDNA for the last few years, although it has been the cDNA routinely used by our research group, and the only one appearing in the few publications regarding *Dlk2 *[1,29]. Thus, our first objective was to confirm or refute if BC019431 was indeed a real cDNA, and to analyze which of the three mRNAs assigned to *Dlk2 *are expressed in different mouse tissues and cell lines. Considering the differences among the mRNAs, and to facilitate the nomenclature of the transcripts, we classified them in two groups, according to the position of their TSS: a first group, including BC118057 and BC122518, was named as variant 1, (V1, Figure 1A); a second group, including only BC019431, was named variant 2 (V2, Figure 1A). We next designed specific oligonucleotides capable to discriminating between V1 and V2 (Figure 1A), and studied by RT-PCR whether any, or both *Dlk2 *mRNA variants described above were present in total RNAs from different origins. We prepare cDNA from total RNA extracted from heart, spleen, testis, brain, and lung of adult 129/C57 mice. The rest of the cDNAs were prepared separately from different mouse cell lines in which *Dlk2 *is expressed, including 3T3-L1, NIH3T3, C3H10T1/2 and AT3F cells [1] (see Materials and Methods). Lastly, mouse genomic DNA was used as a control for primer amplification. As shown in Figure 1B, both PCR primer sets were able to amplify the expected DNA fragments from the genomic DNA sample. However, when cDNA was used as a template, either from tissues or from each cell line, only the V2_U_/V_L _oligonucleotide pair could amplify a fragment of the expected size from all the samples (Figure 1B). The V1_U_/V_L _oligonucleotide pair could not amplify a DNA fragment of the expected size in any of the nine samples analyzed. However, when brain cDNA was used as a template, and the PCR conditions were pushed to try to amplify any trace cDNA, a faint 700 bp fragment could be detected (Figure 1B). Our data thus indicated that only the V2 transcript can be detected in all the samples analyzed, suggesting that clone BC019431 derives from a real mRNA and it is not an artifact. It can also be concluded that V1 transcripts, as they have been described in the genomic databases, are not present in the tissues and cell lines tested in this work or, if they are, their abundance is much lower than that of the V2 transcript. It is interesting to note the possible existence of a different, yet uncharacterized, splice variant that can only be detected in brain, which we plan to analyze in detail in future studies.

To further characterize the *Dlk2 *mRNA, we next focused on mapping the TSS. We first analyzed the expression level of *Dlk2 *in several cell lines, and decided to use the mouse hepatoma cell line AT3F as a source of RNA, due to its high level of *Dlk2 *expression (Figure 1C). We used the Rapid Amplification of cDNA Ends assay (SMART RACE cDNA amplification kit, Clontech, EEUU) to map the 5' region of *Dlk2 *mRNA. Using a *Dlk2 *specific oligonucleotide, we amplified a unique 880 bp DNA band (Figure 1D). This PCR product was next cloned into the vector pCR2.1 (Invitrogen) and twenty independent clones were selected for DNA sequencing. Our sequence data first showed that all clones contained exons that were only compatible with the V2 variant and, therefore, also with the exon-intron distribution of the cDNA clone BC019431 (Figure 1D). In addition, most of the clones (75%) contained 14 additional bases as a 5' extension when compared to the sequence of BC019431, and the remaining 25% contained shorter 5' extensions. The complete *Dlk2 *mRNA sequence, which starts at an A located 203 bases upstream of the ATG start of protein translation, has been submitted to Genbank with the entry number FM180474 [GenBank: FM180474].

Taken together, our data indicate that in the tissues and cell lines analyzed, the only *Dlk2 *mRNA species that could be detected is solely compatible with the cDNA clone BC019431 and, at least in the AT3F cell line, that mRNA possesses a 14 bp 5' extension as compared to this clone. Once the TSS was located, our data also suggested that the sequences in charge of controlling *Dlk2 *gene expression were probably placed in the surroundings of exon E1b (Figure 1A).

### Transcriptional analysis of the *Dlk2 *promoter region

**Figure 2:**
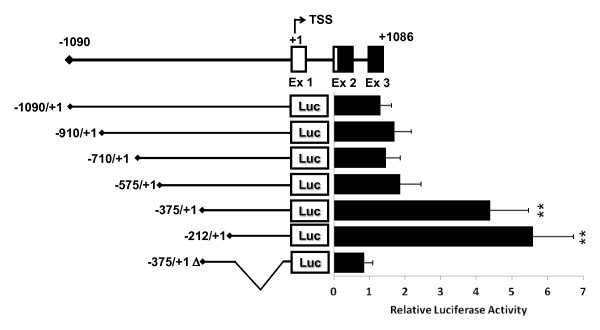
**Characterization of an activator and a repressor sequences in the *Dlk2 *promoter**. NIH3T3 cells were transiently transfected with luciferase constructs encompassing different regions of the *Dlk2 *promoter cloned into pGL3Basic vector, along with the *Renilla *pRL-TK plasmid. Luciferase activity was measured 24 hours after transfection, and each luciferase value (Relative Light Units) was normalized to its corresponding value of *Renilla *activity. The average values of at least three independent experiments are shown. (*, ** and ***, significant *versus *control in Student's *t*-test with p-values < 0.05, < 0.01 and < 0.001, respectively).

We decided to analyze the basal transcriptional regulation of the *Dlk2 *promoter region around 1 Kbp upstream of the TSS. To do that, we cloned a 1,090 bp DNA fragment spanning from position -1,090 to +1 (+1 corresponds to the TSS) into the pGL3Basic vector. We also made a series of constructs in the same vector, containing different 5' deletions of that fragment (Figure 2). All the constructs were tested for their transcriptional activity by transfecting them into NIH3T3 cells and measuring their corresponding induced luciferase activity. All the luciferase measurements were normalized against the *Renilla *activity of the pRL-TK vector. Our results showed that the largest fragment, -1,090/+1, displayed a very low transcriptional activity, as it happened also with the first three deletions studied, -910/+1, -710/+1, and -575/+1 (Figure 2). Surprisingly, the maximum luciferase activity of the *Dlk2 *promoter region was obtained with the fragments -375/+1 and -212/+1, corresponding to deletions eliminating most of the 5' region of the longest fragment, indicating that the core promoter is located within those fragments. To confirm the position of the core promoter, we made an internal deletion in clone -375/+1, in the vicinity of the TSS (positions -197 to -18), that we called -375/+1Δ. As shown in Figure 2, the internal 179 bp deletion in the -375/+1Δ DNA fragment caused a marked decrease in its basal transcriptional activity. Our data allowed the location of the minimal DNA sequence with promoter activity in the -212 to +1 region, and showed that the sequence located between -197 and -18 plays an important role in the regulation of *Dlk2 *transcription.

The fact that longer DNA fragments showed lower transcriptional activity than shorter fragments suggested the existence of transcriptional inhibitory sequences located between positions -1,091 and -375. To test that hypothesis, we decided to use the pGL3Promoter vector, which shows a higher basal transcriptional activity due to the presence of the strong SV40 promoter. We generated four deletions of the fragment -1,090/-375, which were cloned into pGL3Promoter, and we tested their transcriptional activity in NIH3T3 cells. As shown in Additional file 1, Figure S1, all constructs showed a statistically significant reduction of around 30% in their luciferase activity when compared to the pGL3Promoter control vector, thus indicating the existence of sequences able to repressing the transcriptional activity of the SV40 promoter. At this stage we decided to check the publically available genomic map of chromatin modifications performed in cells with different degrees of differentiation [30]. In that map, the methylation of certain lysine residues of Histone H3 (K4, K9, K20 and K27) is mapped, and is correlated with the activity of the promoter. According to that map, *Dlk2 *presents two regions with H3 methylation corresponding to repressed chromatin: one with exclusive H3K27me3 methylation in the region -1,502/-233, and another one with double methylation H3K27me3 (+484/+1232) and H3K4me3 (+382/+895). Interestingly, the inhibitory region we have mapped (-1,090/-375) is located within the first repressor region cited. Therefore, this first analysis of the *Dlk2 *promoter region allowed us to conclude that the minimal region with transcriptional activity is located between positions -212 and +1, and that repressor sequences are located between positions -1,090 and -375.

**Figure 3:**
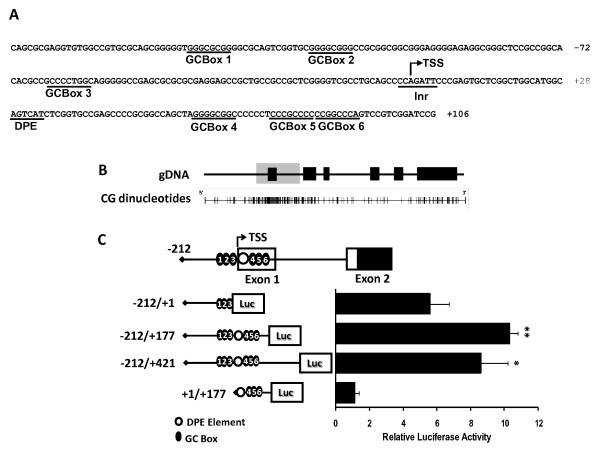
**Identification of the *Dlk2 *core promoter**. A) Core promoter elements in the *Dlk2 *promoter sequence: Initiator element (Inr), Downstream Core Promoter Element (DPE), and six GC-boxes, putative binding sites for Sp1 transcription factor. B) Schematic representation of the CpG island present in the *Dlk2 *promoter, between positions -481 and +440. The grey square represents the localization of the CpG island; black lines represent GC dinucleotides, and dark squares represent *Dlk2 *exons. C) NIH3T3 cells were transiently transfected with pGL3Basic(-212/+1), pGL3Basic(-212/+177), pGL3Basic(-212/+421), or pGL3Basic(+1/+177), along with pRL-TK. Luciferase activity was measured 24 hours after transfection, and each luciferase value (Relative Light Units) was normalized to its corresponding value of *Renilla *activity. The average values of at least three independent experiments are shown. (*, ** and ***, significant *versus *control in Student's *t*-test with p-values < 0.05, < 0.01 and < 0.001, respectively).

We next performed a bioinformatics analysis of *Dlk2 *promoter region that showed the absence of a consensus TATA box and the existence of a potential Initiator sequence (Inr), YYANWYY (where Y is a pyrimidine, N is any nucleotide, and W is adenine or thymine) [31-33] between the bases -2 and +4 around the TSS. A Downstream Core Promoter Element (DPE), whose consensus sequence is RGWYVT (where R is a purine, and V is guanine or adenine or cytosine) [33-35], was also identified in the *Dlk2 *promoter between bases +28 and +33 (Figure 3A). Interestingly, a CpG island was also detected between positions -481 and +440, which extends from the putative core promoter to the first intron, including the non-coding first exon (Figure 3B). Therefore, *Dlk2 *appears to be a gene with a TATA-less promoter associated to a CpG island and, as it happens with other genes with that type of promoter, it also features the presence of GC-boxes. Six GC boxes, potential binding sites for the transcription factor Sp1, were detected in the region close to the TSS, between positions -160 and +90 (Figure 3A). In the absence of a TATA box, Sp1 appears to be involved in the formation of the pre-initiation complex (PIC) and in the transcriptional activation, in conjunction with the Inr element [33,36-39].

The fact that there were putative Sp1 binding sites downstream of the DPE consensus sequence made us consider the idea that transcriptional regulatory regions could be located downstream of the TSS. To explore this, we cloned into pGL3Basic several DNA fragments spanning the region located between bases -212 and +421, from the start of the core promoter to part of the first intron; those plasmids were transfected into NIH3T3 cells and their transcriptional activity was analyzed (see Methods). DNA fragment -212/+177, which contains the full core promoter region (-212/+1), the Inr element, the DPE element, and all putative Sp1 binding sites, caused a significant increase in luciferase activity as compared to fragment -212/+1 (Figure 3C). This indicated the presence of additional activating sequences in that region. The transcriptional activity of fragment -212/+427 was very similar to that of fragment -212/+177, indicating the absence of additional activating sequences in the part of the first intron located between bases +177 and +427 (Figure 3C). Taken together, our data indicate that, although the minimal promoter sequence is located in fragment -212/+1, additional sequences contributing to increase the level of basal transcriptional activity of the *Dlk2 *promoter are present in fragment +1/+177. According to those data, the *Dlk2 *core promoter comprises the Inr and DPE elements, along with several binding sites for the Sp1 transcription factor, located between positions -160/-100 and +52/+92.

### Sp1 activates the *Dlk2 *promoter

**Figure 4:**
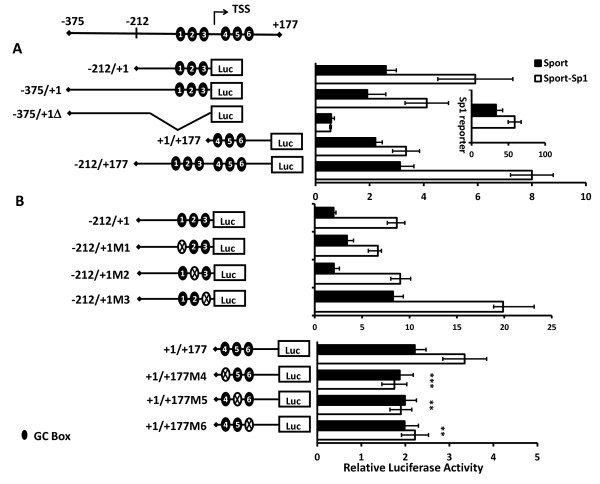
**Sp1 transactivates the *Dlk2 *promoter**. NIH3T3 cells were transiently transfected with A) pGL3Basic(-212/+1), pGL3Basic(-375/+1), pGL3Basic(-375/+1Δ), pGL3Basic(+1/+177), or pGL3Basic(-212/+177); or B) pGL3Basic(-212/+1), pGL3Basic(-212/+1M1), pGL3Basic(-212/+1M2), pGL3Basic(-212/+1M3), pGL3Basic(+1/+177), pGL3Basic(+1/+177M4), pGL3Basic(+1/+177M5), or pGL3Basic(+1/+177M6), along with either pCMVSport (control) or pCMVSport-Sp1, to drive the overexpression of Sp1. A Sp1 luciferase-reporter plasmid was used as a control of Sp1 activity. Luciferase activities were measured 24 hours after transfection. In each transfection, luciferase values were normalized to the corresponding *Renilla *values, as previously described. The average values of at least three independent experiments are shown. (*, ** and ***, significant *versus *control in Student's *t*-test with p-values < 0.05, < 0.01 and < 0.001, respectively).

To explore whether Sp1 regulates *Dlk2 *transcription, and which of the six Sp1 putative binding sites could be involved in that process, we co-transfected into NIH3T3 cells different *Dlk2 *promoter-luciferase constructs containing the potential Sp1 binding sites, along with a plasmid expressing Sp1 (pCMVSport-Sp1), or the empty vector (pCMVSport) as a control. To preserve the total amount of DNA transfected into the cells, in these experiments we used half the amount (0.4 μg) of reporter plasmid as compared to previous experiments (see Methods). Our results showed that all promoter fragments containing Sp1 binding sites, -212/+1, -375/+1, -212/+177, and +1/+177, induced luciferase activity when Sp1 was overexpressed (Figure 4A); on the other hand, the fragment -375/+1Δ, which lacks any Sp1 binding site, showed no responsiveness to Sp1.

To analyze the involvement of each GC box in the induction of transcriptional activity, we next mutated each one of them separately in their corresponding fragments, and tested whether the mutations affected the way they responded to Sp1. As shown in Figure 4B, when we analyzed the activity of the mutant Sp1 binding sites located upstream of the TSS (binding sites 1, 2 and 3), we could not detect any significant variation in the way the different mutants responded to Sp1. However, mutations of the Sp1 binding sites 4, 5 and 6, located in the +1/+177 fragment, led to a significant reduction of their responses to Sp1 in all cases (Figure 4B). Our results demonstrate the existence of functional Sp1 response elements within the first exon of *Dlk2*, and possibly in its proximal promoter, that might transcriptionally regulate the expression of *Dlk2*.

### Sp1 specifically binds to the *Dlk2 *promoter

**Figure 5:**
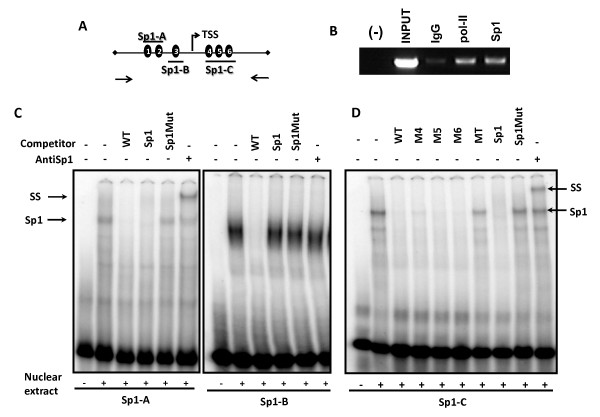
**Sp1 binds to the *Dlk2 *promoter**. A) Schematic representation of the six GC-boxes in the *Dlk2 *promoter, the putative Sp1 binding sites (named 1, 2, 3, 4, 5, and 6). The oligonucleotides used for the ChIP analyses (arrows), and the approximate positions of three oligonucleotides used in EMSA assays are also shown. Oligonucleotide Sp1-A contains the GC boxes located at positions -130/-140 (GCBox-1); -108/-115 (GCBox-2); oligonucleotide Sp1-B contains the GC box located at position -61/-70 (GCBox-3); and oligonucleotide Sp1-C contains the GC boxes located at positions +62/+68 (GCBox-4); +76/+82 (GCBox-5); and +81/+90 (GCBox-6). B) Chromatin IP analysis was performed using native chromatin from 3T3-L1 cells, incubated with normal rabbit IgG (IgG), with antibodies against RNA-polymerase II (pol-II), or with antibodies against Sp1. The PCR analysis and the corresponding agarose gel electrophoresis of a representative experiment is shown. C) and D) EMSA analyses were performed using 8 μg of nuclear protein extracts from NIH3T3 cells, which were incubated with the ^32^P-labeled oligonucleotides Sp1-A, Sp1-B or Sp1-C. For competition and super-shift assays, the reaction was preincubated with a 100-fold excess of the indicated cold oligonucleotides, or with 2 μg of Sp1 antibody before the addition of the labeled oligonucleotide. The locations of the Sp1 and Sp1 supershifted (SS) bands are indicated by arrows.

To study whether Sp1 directly binds to the *Dlk2 *promoter, we next performed chromatin immunoprecipitation analyses (ChIP) in 3T3-L1 cells with the ChIP-IT Express Kit (Active Motif). We used normal rabbit IgG as a negative control, and antibodies against RNA-polymerase II as a positive immunoprecipitation control. We also performed immunoprecipitation with an antibody against Sp1 (PEP2, Santa Cruz Biotechnology Inc.). Purified DNA from the immunoprecipitated samples was used as template for PCR, using a pair of primers specific to *Dlk2 *(indicated by arrows in Figure 5A), which amplify the region surrounding the TSS (see Materials and Methods). The results (Figure 5B) indicated that Sp1 directly binds to the *Dlk2 *promoter region, and suggested that Sp1 can be directly responsible of the regulation of *Dlk2 *expression reported in previous studies.

To further analyze Sp1 binding to *Dlk2*, we next explored by EMSA which of the six GC-boxes were directly bound by that transcription factor. We designed three double-stranded oligonucleotides, containing one, two or three of the six GC Boxes (Figure 5A), that were radiolabeled and incubated with nuclear protein extracts from NIH3T3 cells expressing Sp1. Following electrophoresis and autoradiography, we detected the formation of DNA-protein complexes with the three oligonucleotides tested (Figure 5C and 5D). These complexes appeared to be specific in the case of oligonucleotides Sp1-A and Sp1-C, since they could be competed out both by a 100-fold excess of the unlabeled oligonucleotide, and by a similar excess of a commercial consensus Sp1 oligonucleotide. In addition, the same commercial oligonucleotide in which the Sp1 consensus site had been mutated was unable to compete with the labeled oligonucleotides. Finally, the addition of a specific antibody against the Sp1 protein produced a supershift in both cases. We also detected a protein-DNA complex with the Sp1-B oligonucleotide, but the competition and supershift assays were negative.

As oligonucleotide Sp1-C contains three of the six GC-boxes (4 through 6), we synthesized a series of mutated versions of this oligonucleotide; three of them in which each one of the Sp1 binding sites was individually mutated (oligonucleotides M4, M5 and M6); and one in which the three sites were simultaneously mutated (oligonucleotide MT). When these oligonucleotides were used as binding competitors to Sp1 in EMSA analyses, we found that only MT was unable to block the formation of the Sp1-DNA complex (Figure 5C). The fact that the single mutations did not abolish the competing capacity of each oligonucleotide indicates that Sp1 binds at least to two out of the three GC boxes present in the oligonucleotide Sp1-C.

Our results point to Sp1 as an important regulator of *Dlk2 *transcription, able to bind to two different regions of the *Dlk2 *promoter, one located between positions -100 and -160, and the other between positions +52 and +92.

### Downregulation of Sp1 expression by siRNA results in direct downregulation of *Dlk2*

**Figure 6:**
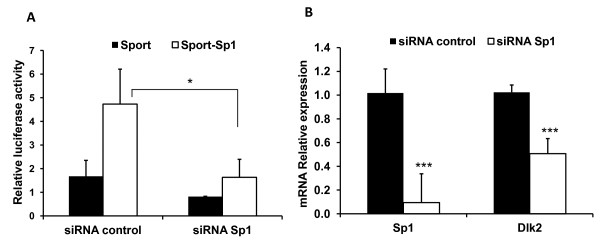
**siRNA specific to Sp1 reduces the transcriptional activity of the *Dlk2 *promoter and the expression of its mRNA**. A) NIH3T3 cells were transiently transfected with the luciferase construct pGL3Basic(-212/+427), along with either pCMVSport (control) or pCMVSport-Sp1, to drive the overexpression of Sp1, and with a Sp1 siRNA or its negative control. Luciferase activities were measured 48 hours after transfection. In each transfection, luciferase values were normalized to the corresponding *Renilla *values, as previously described. B) NIH3T3 cells were transiently transfected with 10 nM of a Sp1-specific siRNA or with the corresponding negative control (Life Technologies). 48 hours after transfection the Sp1 and *Dlk2 *mRNA expression levels were measured by RT-qPCR. mRNA levels were referred to the expression level of phosphoriboprotein P0, used as an internal control, and the normalized values were referred to the values obtained with the negative control. The average values of three independent experiments are shown. (*, ** and ***, significant *versus *control in Student's *t*-test with p-values < 0.05, < 0.01 and < 0.001, respectively).

To provide additional support about the role of Sp1 on the regulation of *Dlk2 *expression, we carried out two sets of RNA interference experiments. We first analyzed the effect of the downregulation of *Sp1 *on the transcriptional activity of a luciferase reporter plasmid carrying the six putative Sp1 binding sites present in *Dlk2 *promoter. For that, NIH3T3 cells were transiently co-transfected either with pGL3basic-212/+427, with a *Silencer Select *siRNA specific to Sp1 (Life Technologies), or with the corresponding negative control, along with the Sp1 expression plasmid, pCMVSport-Sp1, or its corresponding empty vector. As shown in Figure 6A, transfection with *Sp1 *specific siRNA resulted in a reduction of both basal activity, and Sp1 mediated activation of the reporter plasmid, demonstrating the existence of an Sp1 responsive element in the *Dlk2*promoter.

To fully demonstrate the role of Sp1 in *Dlk2 *regulation, we next performed transient transfections in NIH3T3 cells, either with the Sp1 siRNA or with its negative control, and analyzed the effects of Sp1 downregulation on *Dlk2 *transcription by RT-qPCR. As shown in Figure 6B, the reduction of Sp1 expression resulted in a 50% reduction in the amount of *Dlk2 *mRNA, indicating that Sp1 plays an important role in the regulation of *Dlk2 *expression.

## Discussion

The work presented here adds new elements to understanding the biology of *Dlk2*, providing new insights into the molecular mechanisms that contribute to its expression. The mouse *Dlk2 *genomic locus has been constantly revised during the last years, which has led to frequent changes in the assignment of the corresponding predicted transcripts. At present, the sequence of the mouse *Dlk2 *locus appears as definite, but the number of *Dlk2 *putative transcripts varies among databases. In the RefSeq database, there were three full-length mRNAs associated to *Dlk2*, BC118057, BC122518, and BC019431. In this work, we have identified, in all tissues and cell lines tested, a unique *Dlk2 *transcript, only compatible with cDNA clone BC019431. Our directed PCR analyses and the results of the RACE assays showed that the TSS identified for *Dlk2 *is only compatible with that transcript (Figure 1B). Our results also show a 14 bp 5' extension of the seemingly truncated BC019431 clone. A new entry taking into consideration our experimental data has been established in *GenBank/EMBL/DDBJ *databases, with ID FM180474. As mentioned above, the BC019431 entry was temporarily eliminated from the databases, and even today it is still defined as a *chimeric *clone. However, in the variety of mouse tissues and cell lines used for the characterization of *Dlk2 *transcripts in this work we have reported the existence of a single major *Dlk2 *mRNA species consistent with clone BC019431. Interestingly, we have also detected a minority transcript, yet uncharacterized, that seems to be expressed only in the brain, and that does not correspond to any of de V1 transcripts previously described. Although we cannot rule out the existence of additional mRNA transcripts expressed in other tissues or at different developmental stages, our data show that clone BC019431, and its extended version, clone FM180474, are clearly *non-chimeric *mRNAs.

Analysis of the *Dlk2 *promoter transcriptional activity by luciferase assays revealed that the shortest fragment with transcriptional activity is located between positions -212 and +1, and that the deletion of 179 bp located between positions -194 and -18 led to the complete abolition of *Dlk2 *transcriptional activity (Figure 2). Unexpectedly, the largest fragment tested, -1,090/+1, showed very low transcriptional activity, apparently due to the presence of inhibitory sequences in the region located between -1,090 and -375 bp, as confirmed by luciferase assays performed with strong SV40 promoter constructs. It seems that the *Dlk2 *promoter shows low level of basal transcription, due to the mentioned repressive sequences. We have explored the predicted state of the chromatin at the *Dlk2 *promoter region, taking advantage of the publically available genomic map of chromatin modifications performed in cells with different degrees of differentiation, focused on the methylation of certain lysine residues of these proteins, such as K4, K9, K20 and K27 of Histone H3 [30]. According to this map, *Dlk2 *presents two regions with H3 methylation, the first one with H3K27me3 methylation (region -1,502/-233), in ES cells and in MEFs, and the second one with double H3K4me3, H3K27me3 methylation (+484/+1232 and +382/+895) in ES cells (http://www.ensembl.org). According to the authors of the map, single methylation at residue K4 is associated with an active chromatin state; however, methylation at residue K27 is a mark of repressed chromatin. Some promoters present simultaneously both H3K4 and H3K27 methylation, and in most of them the basal transcription is repressed [30]. These ambiguous promoters have been associated to genes of complex expression, including cell surface molecules and key transcription factors, during development or morphogenesis. The first H3K27me3 region coincides with the repressor region mapped in our luciferase experiments, and could be in part responsible for the basal repressed state of *Dlk2*, a membrane protein involved in morphogenesis, developmental and differentiation processes [1].

Bioinformatics analyses showed that *Dlk2 *is a TATA-less promoter gene, as are about 74% of human genes [33,39,40]. That analysis also revealed the existence of core promoter consensus elements, such as Inr and DPE. Those elements are common in TATA-less promoters: they are required to trigger transcription in the absence of a TATA sequence [31,41]. An important aspect of the *Dlk2 *promoter structure is the presence of a CpG island between positions -481 and +440, which extends from the putative core promoter to the first intron, including the non-coding first exon. CpG islands are potentially sensitive to DNA-methylation, and could participate in gene transcriptional regulation [42]. The basal state of CpG islands in the promoters possessing them is non-methylated, although methylation can occur at certain times during development, to achieve specific gene silencing [43-46]. So far, nothing is known about the methylation state of the *Dlk2 *CpG island. This issue is, however, of great potential importance for the understanding of the transcriptional regulation of *Dlk2 *during development, and along the differentiation processes in which that gene participates, and is currently under study in our laboratory.

When TATA-less promoters are associated to CpG islands generally they contain consensus GC-boxes in the region close to the TSS that could be recognized by the transcription factor Sp1 [33,47]. It has been reported that, together with the Inr element, Sp1 can regulate the transcription of those genes [37,38]. There are six GC-boxes in the *Dlk2 *promoter, located between positions -160 and +90. We have shown that the transcription factor Sp1 could bind to the *Dlk2 *promoter in that region, both by ChIP and by EMSA analyses. In addition, our luciferase assays have shown that the activity of the *Dlk2 *promoter is modulated by Sp1, and that mutation of some of the Sp1 binding sites abolished Sp1-mediated *Dlk2 *transactivation. We have also demonstrated that a reduction in the expression level of Sp1, using siRNA technology, results in a decrease of the transcriptional activity of the *Dlk2 *promoter, as well as a reduction in the amount of *Dlk2 *mRNA. Our results indicate, therefore, that Sp1 is a key regulator of *Dlk2 *transcription.

Interestingly, during *in vitro *adipogenic assays in 3T3-L1 cells, Sp1 expression is reduced in response to some of the components of the adipogenic differentiation cocktail, including IBMX, a cAMP phosphodiesterase inhibitor, and the glucocorticoid dexamethasone. Sp1 has also been involved in the control of transcription of several genes that are essential for the correct onset of adipogenesis; an IBMX-dependent reduction of Sp1 expression causes a derepression of the *C/ebpα*gene, thus promoting adipogenesis [48]. Other authors have recently reported that the Sp1-dependent downregulation of the Tissue Inhibitor of Metalloproteinase 3 (TIMP-3) is necessary for adipogenesis [49]. On the other hand, unpublished data from our group shows that *Dlk2 *is an important factor regulating the early stages of adipogenesis, being a transcriptional target of the crucial transcription factor KLF4. *Dlk2 *expression is tightly controlled during the first hours of the adipogenic differentiation, showing a peak of expression two hours after the induction with IBMX, and maintaining a low level of expression during the rest of the process. The fact that the expression of both Sp1 and *Dlk2 *are controlled by the same molecule during early adipogenesis, together with the role of Sp1 in activating the expression of *Dlk2*, raise the interesting possibility that *Dlk2 *could be a transcriptional target of Sp1 during the adipogenesis process. Finally, we have recently described that together with *Dlk1, Dlk2 *acts as a NOTCH signaling regulator [29]. The control of *Dlk2 *expression, therefore, may be a mechanism with important consequences for the regulation of the numerous differentiation processes in with NOTCH receptors participate. Further studies are granted to explore these possibilities.

## Conclusions

We show here the characterization of the mouse *Dlk2 *transcript in several preadipocitic and hepatoma cell lines, as well as in adult brain, spleen, heart, liver, and testis. In all the samples the transcript appears as a unique species, with a 14 bases 5' extension related to the previously described clone BC019431, and it has been entered in GenBank with the ID [GenBank: FM180474]. *Dlk2 *possesses a TATA-less promoter, with the consensus sequences Inr and DPE, and located within a CpG island. There are sequences able to repress transcription, located at position -1,090/-375 that may in part be responsible for the repressed basal state of the *Dlk2 *promoter. The minimal sequence with transcriptional activity is located between positions -212 and +1. The *Dlk2 *core promoter contains six GC-boxes between the position -160 and +92, consensus sequences for the binding of the transcription factor Sp1. We have shown, both by ChIP and by EMSA analyses that Sp1 binds to the *Dlk2 *promoter in that region. Additionally, we have demonstrated that the activity of the *Dlk2 *promoter is modulated by Sp1, and that mutation of some of the Sp1 binding sites abolished Sp1-mediated *Dlk2 *transactivation. Our results indicate, therefore, that Sp1 could be a key regulator of *Dlk2 *transcription.

## Methods

### DNA constructs

For the analysis of *Dlk2 *transcriptional activity, we cloned by PCR different fragments of its promoter region, using DNA from BAC clone RP23-135A16 (BACPAC Resources, USA) as template, which contains the complete sequence of the *Dlk2 *promoter, and the oligonucleotides indicated in Table 1. PCR reactions were performed under standard conditions, except where indicated with an asterisk, in which case the reactions were supplemented with 10% DMSO. The DNA from the PCR amplification was digested with the restriction enzymes indicated in Table 1, and inserted into the vectors pGL3Basic or pGL3Promoter (Promega, USA). The fragment -375/+1Δ was generated by the amplification of the fragment -375/+1 with the oligonucleotides listed in Table 1 under standard PCR conditions; in the absence of 10% DMSO, the PCR reaction generated a 196 bp fragment (-375 to +1) with a 179 bp internal deletion between positions -197 and -18. DNA from the PCR amplifications was digested with *Mlu*I-*Hind*III, and cloned into the vector pGL3Basic. The mutant luciferase constructs pGL3Basic(-212/+1M1), pGL3Basic(-212/+1M2), pGL3Basic(-212/+1M3), pGL3Basic(+1/+177M4), pGL3Basic(+1/+177M5), and pGL3Basic(+1/+177M6), were generated with the Quick-Change site-directed mutagenesis kit (Stratagene, USA), using the pGL3Basic(-212/+1) or pGL3Basic(+1/+177) constructs as templates. The primers used for mutagenesis are indicated in Table 2. All the constructs were sequence-verified, using 0.5 to 1.0 μg of each plasmid for sequencing with the ABI PRISM dRhodamine Terminator Cycle Sequencing Ready Reaction Kit (Applied Biosystems, Carlsbad, CA, USA). Sp1 expression plasmid (pCMVSport-Sp1) and Sp1 luciferase-reporter plasmid were kindly provided by Dr. Marta Casado-Pinna.

**Table 1:** Oligonucleotides used for the cloning of different fragments of *Dkl2 *promoter in pGL3Basic and pGL3promoter vectors

DNA FRAGMENT	OLIGONUCLEOTIDES	SEQUENCE	RESTRICTION ENZYMES
-1,090/+1*	Dlk2MluI-917U	5'-ATTACGCGTTTGTCAGGTGTAGGCGGTGGG-3'	*Mlu*I-*Hind*III
	Dlk2HindIII-1L	5'-TATAAGCTTGCTGAGGCGACCCCGAGCG-3'	

-910/+1*	Dlk2MluI-722U	5'-GGCGACGCGTCAAATACACATATTGGGGTCTT-3'	*Mlu*I-*Hind*III
	Dlk2HindIII-1L	5'-TATAAGCTTGCTGAGGCGACCCCGAGCG-3'	

-710/+1*	Dlk2MluI-522U	5'-TATACGCGTGGCAGGCTACCCAAAGGTGG-3'	*Mlu*I-*Hind*III
	Dlk2HindIII-1L	5'-TATAAGCTTGCTGAGGCGACCCCGAGCG-3'	

-575/+1*	Dlk2MluI-400U	5'-TAGACGCGTAAGAAGCCCACAGAGAGCAGGC-3'	*Mlu*I-*Hind*III
	Dlk2HindIII-1L	5'-TATAAGCTTGCTGAGGCGACCCCGAGCG-3'	

-375/+1*	Dlk2MluI-205U	5'-TATACGCGTTGGGTGAGGGGCAGAGTGG-3'	*Mlu*I-*Hind*III
	Dlk2HindIII-1L	5'-TATAAGCTTGCTGAGGCGACCCCGAGCG-3'	

-212/+1*	Dlk2 Xho-212U	5'-TATCTCGAGGAAGGGAGGGGCGAAGAGC-3'	*Xho*I-*Hind*III
	Dlk2HindIII-1L	5'-TATAAGCTTGCTGAGGCGACCCCGAGCG-3'	

-1,090/-375	Dlk2MluI-917U	5'-ATTACGCGTTTGTCAGGTGTAGGCGGTGGG-3'	*Mlu*I-*Hind*III
	Dlk2HindIII-184L	5'-GCTAAGCTT CCACTCTGCCCCTCACCCAC-3'	

-1,090/-575	Dlk2MluI-917U	5'-ATTACGCGTTTGTCAGGTGTAGGCGGTGGG-3'	*Mlu*I-*Hind*III
	Dlk2HindIII-379L	5'-TAGAAGCTTCGCCTGCTCTCTGTGGGCTTC-3'	

-910/-375	Dlk2MluI-722U	5'-GGCGACGCGTCAAATACACATATTGGGGTCTT-3'	*Mlu*I-*Hind*III
	Dlk2HindIII-184L	5'-GCTAAGCTT CCACTCTGCCCCTCACCCAC-3'	

-710/-375	Dlk2MluI-522U	5'-TATACGCGTGGCAGGCTACCCAAAGGTGG-3'	*Mlu*I-*Hind*III
	Dlk2HindIII-184L	5'-GCTAAGCTT CCACTCTGCCCCTCACCCAC-3'	

+1/+177	Dlk2Xho I+1U	5'-ATTCTCGAGCCCAGATTCCCGAGTGCTCGGC-3'	*Xho*I-*Hind*III
	Dlk2HindIII+177L	5'-ATGAAGCTTAGGGACCGCGTCCTCCTAGCTTC-3'	

-212/+177*	Dlk2 Xho-212U	5'-TATCTCGAGGAAGGGAGGGGCGAAGAGC-3'	*Xho*I-*Hind*III
	Dlk2HindIII+177L	5'-ATGAAGCTTAGGGACCGCGTCCTCCTAGCTTC-3'	

-212/-427*	Dlk2 Xho-212U	5'-TATCTCGAGGAAGGGAGGGGCGAAGAGC-3	*Xho*I-*Hind*III
	Dlk2HindIII+427L	5'-AATAAGCTT GTGGCCACCGCGCGGGAAC-3	

PCR reactions were performed under standard conditions (see Materials and Methods), except where indicated with an asterisk, in which the reactions were supplemented with 10% DMSO.

**Table 2:** Oligonucleotides used for the mutations of *Dlk2 *GC-boxes

PLASMID	OLIGONUCLEOTIDES	SEQUENCE
pGL3Basic(-212/+1M1)	Sp1212/1M1F	5'- GTGCGCAGCGGGGGTGG**ATATAA**GGCGCAGTCGGTGCGGG -3'
	Sp1212/1M1R	5'- CCCGCACCGACTGCGCC**TTATAT**CCACCCCCGCTGCGCAC -3'

pGL3Basic(-212/+1M2)	Sp1212/1M2F	5'- GGGCGCAGTCGGTGCGG**AATAA**GCCGCGGCGGCGGGAGG -3'
	Sp1212/1M2R	5'- CCTCCCGCCGCCGCGGC**TTATT**CCGCACCGACTGCGCCC -3'

pGL3Basic(-212/+1M3)	Sp1212/1M3F	5'- GGCTCCGCCGGCACACG**TTATT**CCTGGCAGGGGGCCGAG -3'
	Sp1212/1M3R	5'- CTCGGCCCCCTGCCAGG**AATAA**CGTGTGCCGGCGGAGCC -3'

pGL3Basic(+1/+177M4)	Dlk2MluI-400U	5'- CCCGCGGCCAGCTAGG**TTATTC**CCCCCTCCCGCCCCC -3'
	Dlk2HindIII-1L	5'- GGGGGCGGGAGGGGGG**AATAA**CCTAGCTGGCCGCGGG -3'

pGL3Basic(+1/+177M5)	Dlk2MluI-205U	5'- AGGGGCGGCCCCCCTC**AATAA**CCCGGCCCACGTCCGTC -3'
	Dlk2HindIII-1L	5'- GACGGACGTGGGCCGGG**TTATT**GAGGGGGGCCGCCCCT -3'

pGL3Basic(+1/+177M6)	Dlk2 Xho-212U	5'-GCCCCCCTCCCGCCCC**AATTAA**CAGTCCGTCGGATCCG-3'
	Dlk2HindIII-1L	5'- CGGATCCGACGGACTG**TTAATT**GGGGCGGGAGGGGGGCC -3'

### Cell Culture and RT-PCR Analysis

Mammalian cells were cultured at 37°C in a 5% (v/v) CO_2 _humidified atmosphere, in Dulbecco's modified Eagle's medium (DMEM) for 3T3-L1 (ATCC CCL-92.1), C3H10T1/2 (clone 8, ATCC CCL-226) and NIH3T3 (ATCC CRL-1658) cell lines; DMEM-F12 for the AT3F cell line [50]. In all cases, the media contained 10% (v/v) fetal bovine serum (FBS, Biowhittaker), 2 mM L-glutamine (Biowhittaker), 1 Unit/ml penicillin (Biowhittaker), and 1.0 μg/ml streptomycin (Biowhittaker). For RT-qPCR analysis, total RNA was isolated from cells using an RNeasy kit (Qiagen Inc., USA), including a DNAse treatment step to remove potential genomic DNA contamination. First-strand cDNA was prepared in a 20 μl reaction volume from 1 μg of RNA using 0.5 μg of oligo (dT)_18 _and the RevertAid™ H Minus M-MuLV II reverse transcriptase kit (Fermentas, Spain). Real-time PCR was performed in a final volume of 10 μl containing 0.3 μM of each oligonucleotide, 1 μl of cDNA, and the FastStart SYBR Green Master Mix (Applied Biosystem). Reactions were run in triplicate on a 7500 Fast Real-Time PCR System (Applied Biosystems, Carlsbad, CA, USA) with the following conditions: an initial denaturation step at 95°C for 20 seconds, and 40 cycles of 3 seconds at 95°C, followed by 30 seconds at 60°C. Details of the primer sets used are provided in Additional file 1, Table S1. Controls for genomic DNA and primer contamination were routinely performed with non-RT or no template PCR reactions, respectively. Dissociation curves were performed for each set of oligonucleotides to check primer specificity and to confirm the presence of a unique PCR product. Results were analyzed using comparative Ct method using the ribosomal phosphoprotein P0 as a control for amount of cDNA [51]. To estimate PCR efficiencies, standard curves were performed based on 5 serial dilutions of a cDNA stock (a cDNA mixture of all samples collected). Efficiencies (E) were calculated from the slope of curves using the formula E = 10^(-1/slope)^. The efficiencies of all primer sets were between 95 and 100%.

For siRNA downregulation of Sp1, we used the *Silencer Select siRNA *predesigned for Sp1, and the corresponding negative control (Life Technologies). siRNA were transfected at a concentration of 10 nM into NIH3T3 cells, using Lipofectamine 2000 (Invitrogen). After 48 hours RNA was purified, and RT-qPCR was performed.

### Rapid Amplifications of cDNA Ends (RACE)

To map the 5' end of *Dlk2 *gene transcript we used SMART RACE cDNA Amplification Kit (Clontech, Mountain View, CA, USA). Total RNA was isolated from the AT3F cell line with RNeasy Kit (Qiagen Inc. Valencia, CA, USA), which was used to synthesize cDNA with the oligonucleotides 5 CDS: 5'-(T)_25_VN-3 '(N = A, C, G or T, V = A, G or C), and SMART II A: 5'-AAG CAG TGG TAT CAA CGC AGA GTA CGC GGG-3'. The cDNA was used as a template for PCR, using the primers UPM (Universal Prime Mix): 5'-CTA ATA CGA CTC ACT ATA GGG CAA GCA GTG GTA TCA ACG CAG AGT-3' and 5'RACEDlk2: 5'- CTG GCA TGG GCG GCT GGC ACA GTC ATC CA-3'. The amplified DNA fragments were cloned into the vector pCR2.1 (Invitrogen, CA, USA) and 20 clones were sequenced with the oligonucleotide 5'-TAA CCC GGG GGA TCC ACC AGT GAC CAA GGA-3'.

### Analysis of *Dlk2 *mRNA transcripts

To analyze the expression of *Dlk2 *mRNA transcripts, total RNA was isolated from heart, spleen, testis, brain, and lung of adult 129/C57BL6 mice, using the reagent TRI-Reagent (Sigma, USA), according to manufacturer's instructions. The isolated RNA was purified with the RNeasy Kit (Qiagen Inc. Valencia, CA, USA). Total RNA was also isolated from 3T3-L1, NIH3T3, C3H10T1/2 and AT3F cell lines with the RNeasy kit. cDNA was synthesized from 1.0 μg of total RNA, using the "Reverse H Minus First Strand cDNA Synthesis kit (Fermentas, Spain). These cDNAs were used as templates in PCR reactions under the following conditions: an initial denaturation step at 95°C for 5 minutes, and 45 cycles of 30 seconds at 95°C, followed by 30 seconds at 58°C and 120 seconds at 72°C. The primers used were: V1_U_: GGA GAG CCG GGA AAG GCT AAT G; V2_U_: TCG GCT GGC ATG GCA GCT ACT T; and V_L_: TCA CAC AGC GCT CAC AGT GCA G.

### Luciferase reporter assays

NIH3T3 cells at 80-90% confluence were plated in 24-well plates and transfected using Fu-gene HD reagent (Roche Applied Science, USA). In the experiments performed only with the luciferase reporter plasmids, 0.8 μg of DNA per well were used. However, in the experiments where the luciferase plasmids were transfected along with the Sp1 expression plasmid or the corresponding empty vector, 0.4 μg of each plasmid were used. The pRL-TK (Promega, USA), carrying the *Renilla *luciferase under the control of the thymidine kinase promoter, was also co-transfected as an internal control for transfection efficiency. Cells were harvested 24 hours after transfection and luciferase activities were analyzed using the Dual-Luciferase assay kit (Promega, USA) and MLX Microtiter Plate Luminometer (Dynex Technologies, USA), as recommended by the manufacturer. Luciferase activity was normalized to the *Renilla *activity measured in the same lysate.

### Chromatin immunoprecipitation (ChIP) assays

ChIP analysis of 3T3-L1 cells was performed using ChIP-IT Express Kit (Active Motif, USA), following the manufacturer's recommendations. Twenty million 3T3-L1 cells were formaldehyde-cross-linked, and DNA was sheared by sonication using a Bioruptor Sonication System (Diagenode, Belgium) for 30 minutes. The sheared chromatin was incubated with 3 μg of normal rabbit IgG, or the corresponding antibodies against RNA-polymerase II, and Sp1 (PEP2) (Santa Cruz Biotechnology Inc., USA). IPs were performed with the magnetic beads included in the ChIP-IT Express kit. For PCR, 5 μl of the 100 μl total immunoprecipitated DNA were analyzed using GC-Rich PCR system (Roche Applied Science, USA), with the oligonucleotides Dlk2MluI-212U, 5'-TAT ACG CGT GAA GGG AGG GGC GAA GAG C-3' and Dlk2HindIII+177L, 5'- ATG AAG CTT AGG GAC CGC GTC CTC CTA GCT TC-3'.

### Nuclear extracts and electrophoretic mobility shift assay

For nuclear extracts NIH3T3 cells were washed twice with ice-cold PBS, then scraped and homogenized for 15 min. on ice with hypotonic lysis buffer (10 mM Hepes, pH 8.0; 10 mM KCl, and 1.0 μg/ μl protease inhibitor cocktail from Sigma, USA). IGEPAL (0.5%) was added and the mixture was vortexed for 30 seconds at 4°C, followed by centrifugation at 13,500 r.p.m. for 30 seconds. The nuclear pellets were incubated and vortexed for 30 min. at 4°C with 20 mM HEPES, pH 8.0, 0.4 M NaCl, 20% glycerol and 1.0 μg/ μl of the protease inhibitor cocktail. The samples were centrifuged at 13,500 r.p.m. for 10 min., and the supernatants, containing the nuclear fraction, were collected. EMSA was performed by incubating 8 μg of nuclear extracts in a 20 μl binding reaction mixture containing 10 mM HEPES pH 8.0, 80 mM KCl, 1 mM DTT, 5% glycerol, 0.1 μg/ μl BSA, 0.4 mM MgCl_2_, 2 μM ZnSO_4_, 0.02% IGEPAL, 1 μg poly (dI-dC), and 40,000 cpm of ^32^P-labeled double-stranded DNA probe for 20 min. at room temperature. Following incubation, reaction mixtures were loaded and electrophoresed on a 6% polyacrylamide gel, and subjected to autoradiography. A 100-fold molar excess of unlabeled probe was added for competition when indicated in the corresponding figures. Sp1 and Sp1 mutant oligonucleotides were purchased from Santa Cruz Biotechnology. For competition and supershift experiments, proteins were preincubated with unlabeled probe or with the anti-Sp1 antibody (Sp1(PEP-2), Santa Cruz Biotechnology) for 1 hour at 4°C. Labeled probe was then added, and incubated for 20 min. at room temperature. Gel shift assay oligonucleotide sequences are indicated in Table 3.

**Table 3:** Oligonucleotides used for EMSA

OLIGONUCLEOTIDE		SEQUENCE
***Sp1-A***	**Sp1-As**	5-GCTCCGCCGGCACACGCCGCCCCTGGCAGGGGGCCGAGCGC-3
	**Sp1-Aas**	5-GCGCTCGGCCCCCTGCCAGGGGCGGCGTGTGCCGGCGGAGC-3

***Sp1-B***	**Sp1-Bs**	5-TGCGCAGCGGGGGTGGGCGCGGGGCGCAGTCGGTGCGGGGCGGGCCGCGGCGGCGGGAGG-3
	**Sp1-Bas**	5-CCTCCCGCCGCCGCGGCCCGCCCCGCACCGACTGCGCCCCGCGCCCACCCCCGCTGCGCA-3

***Sp1-C***	**Sp1-Cs**	5-GGCCAGCTAGGGGCGGCCCCCCTCCCGCCCCCCGGCCCAG-3
	**Sp1-Cas**	5-GTGGGCCGGGGGGCGGGAGGGGGGCCGCCCCTAGCTGGCC-3

***M4***	**M4s**	5-GGCCAGCTAGTTTATTCCCCCCTCCCGCCCCCCGGCCCAG-3
	**M4as**	5-CTGGGCCGGGGGGCGGGAGGGGGGAATAAACTAGCTGGCC-3

***M5***	**M5s**	5-GGCCAGCTAGGGGCGGCCCCCCTAAATAACCCCGGCCCAG-3
	**M5as**	5-CTGGGCCGGGGTTATTTAGGGGGGCCGCCCCTAGCTGGCC-3

***M6***	**M6s**	5-GGCCAGCTAGGGGCGGCCCCCCTCCCGCCCCAATTAAAAG-3
	**M6as**	5-CTTTTAATTGGGGCGGGAGGGGGGCCGCCCCTAGCTGGCC-3

***MT***	**MTs**	5-GGCCAGCTAGTTTATTCCCCCCTAAATAACCAATTAAAAG-3
	**MTas**	5-CTTTTAATTGGTTATTTAGGGGGGAATAAACTAGCTGGCC-3

***Sp1 ***	**Sp1s**	5-ATTCGATCGGGGCGGGGCGAGC -3
	**Sp1as**	3-TAAGCTAGCCCCGCCCCGCTCG-5

***Sp1Mut***	**Sp1Muts**	5-ATTCGATCGGTTCGGGGCGAG C-3
	**Sp1Mutas**	3-TAA GCTAGCCAAGCCCCGCTCG-5

Sp1 consensus (Sp1) and Sp1 mutant consensus (Sp1Mut) oligonucleotides were purchased from Santa Cruz Biotechnologies.

## Authors' contributions

SR carried out the molecular analysis of the promoter, the luciferase assays and the EMSAs, and helped to draft the manuscript. ARG participated in the Sp1 luciferase studies. MJMDG participated in the EMSAs and helped with the design of the study. JL initiated and participated in the design of the study and helped to draft the manuscript. JJGR conceived the study, carried out the ChIP analysis and the siRNA studies, designed and coordinated most of the experiments and helped to draft the manuscript. All authors read and approved the final manuscript.

## Supplementary Material

Additional file 1 **Table S1. Primers used for RT-qPCR assays**. Figure S1. Characterization of a repressor sequence in the *Dlk2 *promoter. Click here for file
