# Supplementary material for: Characterization of a proximal Sp1 response element in the mouse Dlk2 gene promoter
Source: BMC Mol Biol. 2011 Dec 20;12:52. doi: 10.1186/1471-2199-12-52 (PMC3296630; doi:10.1186/1471-2199-12-52)
Supplement: Additional file 1 — Table S1. Primers used for RT-qPCR assays. Figure S1. Characterization of a repressor sequence in the Dlk2 promoter. [file 1471-2199-12-52-S1.DOCX]

**TABLE S1: Primers used for RT-qPCR assays.**

| **Gene** | **GeneBank Accesion number** | **Oligonucleotide sequence** | **Exon** | **Amplicon size** |
| --- | --- | --- | --- | --- |
| **Dlk2** | NM_207666 | 5′-ACTGTGAGGTCAATGTGGACGA-3′ | 6 | 76 bp |
|  |  | 5′-CGGTTTATGCCATCAATGCAT-3′ | 6 |  |
| **Sp1** | NM_013672 | 5´- GGCTGCCCATTTGTACTCATTTAC-3´ | 6 | 107 bp |
|  |  | 5´-CCGAAGGGTGCCTGTTAGG -3´ | 6 |  |
| **P0** | NM_007475 | 5′-AAGCGCGTCCTGGCATTGTCT-3′ | 6 | 133 bp |
|  |  | 5′-CCGCAGGGGCAGCAGTGGT-3′ | 7 |  |

|  |
| --- |

**FIGURE S1. Characterization of a repressor sequence in the *Dlk2* promoter.**

**Figure S1.** NIH3T3 cells were transiently transfected with luciferase constructs encompassing different regions of the *Dlk2* promoter cloned into pGL3Promoter vector, along with the *Renilla* pRL-TK plasmid. Luciferase activity was measured 24 hours after transfection, and each luciferase value (Relative Light Units) was normalized to its corresponding value of *Renilla* activity. The average values of at least three independent experiments are shown. (*, ** and ***, significant *versus* control in Student’s *t*-test with p-values <0.05, <0.01 and <0.001, respectively).
